# Study protocol for a peer-led web-based intervention to promote safe usage of dating applications among young adults: a cluster randomized controlled trial

**DOI:** 10.1186/s13063-018-3167-5

**Published:** 2019-02-06

**Authors:** Stephanie Tsz Hei Lau, Kitty Wai Ying Choi, Julie Chen, William Pak-hing Mak, Ho Kong Christopher Au Yeung, Joseph Tucker, William Chi-Wai Wong

**Affiliations:** 1 Department of Family Medicine and Primary Care, Li Ka Shing Faculty of Medicine, The University of Hong Kong, Hong Kong, Hong Kong; 2 VTC School of Business and Information Systems, VTC, Hong Kong, Hong Kong; 3 Department of Social Science, School of Humanities and Social Science, Hang Seng Management College, Hong Kong, Hong Kong; 4 University of North Carolina School of Medicine, University of North Carolina at Chapel Hill, Chapel Hill, USA

**Keywords:** Dating applications, Young adults, Sexual health risks, Cluster randomized controlled trial, Chinese

## Abstract

**Background:**

Dating applications are a popular platform to meet new people. At the same time, they have been associated with risks such as unsafe sexual behavior and privacy concerns in young adults. This paper presents a study protocol of a cluster randomized controlled trial (RCT) to evaluate the effectiveness of a peer-led web-based intervention to promote its safe usage in young adults.

**Methods:**

The study design is an open-labeled cluster RCT with an intervention and a placebo control arm. The intervention group will receive a web-based intervention developed through focus group discussions, a crowdsourcing contest, and a Peer-Vetted Creative Production (PVCP) workshop. The control group will receive a web-based resource on health and exercise.

We aim to recruit approximately 338 young adults aged 17–27 years from three tertiary educational institutions in Hong Kong with the class as the cluster unit. Based on the Information, Motivation, and Behavioral Skills (IMB) model, the primary outcome of this study is self-efficacy in using dating applications measured by the General Self Efficacy Scale. Secondary outcomes include change in risk perception measured by the Risk Propensity Scale and a Risk Assessment Tool. Questionnaires will be administered before the intervention, after the intervention, and at one-month follow-up. Intention-to-treat analysis and multilevel regression modeling will be used to evaluate differences in outcomes between groups and the factors affecting these outcomes, respectively.

**Discussion:**

Dating application usage presents opportunities as well as challenges to young adults meeting new friends. Innovative and relatable interventions are needed to promote the safe usage of dating applications to this population. Practical knowledge gained from the development process may be helpful for future intervention utilizing the peer-led approach. If effective, the intervention will be disseminated to non-governmental organizations and educational institutions to be used as a teaching resource.

**Trial Registration:**

ClinicalTrials.gov, NCT03685643. Registered on 26 September 2018. University of Hong Kong Clinical Trials Registry, HKUCTR-2512.

**Electronic supplementary material:**

The online version of this article (10.1186/s13063-018-3167-5) contains supplementary material, which is available to authorized users.

## Background

With the improvement of mobile technology and reliance on smartphones, the use of dating applications for seeking and initiating relationships has become increasingly prevalent [1]. Dating application users are able to access a wide pool of potential partners who are geographically nearby [2]. Users can meet with mutually interested partners in cyberspace and access a vast network of users at any time of the day. However, many of these applications are free and lack stringent joining criteria, resulting in a diverse range of users and potential dangers [2]. Ultimately, the minimal pre-screening of users combined with low attention to safety considerations can result in users engaging in risky behaviors that could have adverse impacts on health.

Previous studies focusing on the general population and specific subgroups have found associations between dating application usage and adverse health outcomes. A study found that dating application users were significantly more likely to have at least one prior diagnosis of sexually transmitted infection compared to non-users and also had more sexual partners [3]. Homosexual men using dating applications were more likely to have unprotected sexual intercourse [4]. A meta-analysis found that the odds for seroconcordant and serodiscordant unprotected anal intercourse were higher in online-initiated encounters as compared to offline encounters among men-who-have-sex-with-men (MSM) [5]. A study on HIV risk among MSM in East and South-East Asia suggested that online partner-finding websites did not facilitate greater risk-taking but provided more avenues for MSM to engage in risky HIV behavior facilitating the spread of the infection [6].

Several studies have focused particularly on the adverse impact dating applications had on young adults. In a study conducted in Hong Kong college students, dating application use was significantly associated with unprotected sexual intercourse with more lifetime partners, less consistent condom usage, and the likelihood of not using a condom in the last sexual encounter [2]. It is also associated with recreational drug use in conjunction with sexual activities in college students [7]. Another study conducted in university students revealed that dating application users were more likely to have experienced sexual abuse in the past year compared to non-users and at a higher risk for lifetime sexual abuse [8]. The contents presented to adolescents on dating application profiles are also a source of concern. A study analyzing content on a teen-dating site found a variety of risk-related contents such as sex, alcohol and drug use, and violence [9]. Concerns regarding personal information and monetary scams are also prevalent. These studies reveal a strong association between using dating applications and detrimental behavior among young adults, indicating the need for the development of interventions to encourage the safe usage of dating applications in this population.

As adolescents and young adults spend a significant amount of time online, health interventions through the Internet are a promising avenue to reach this population. A systematic review found that interactive computer-based interventions were effective tools for sexual health learning with positive effects on self-efficacy, intention, and sexual behavior [10]. Other advantages of Internet-based interventions is the level of accessibility, confidentially, and availability of tailored messages or content [10].

The peer-led approach as characterized by the “interaction between individuals with shared characteristics such as behavior, experience, status or social and cultural backgrounds” [11], has been shown to be appealing towards health promotion for adolescents and young adults, as individuals in this age group tend to seek advice and information from their peers instead of adults or other figures of authority [12]. Crowdsourcing conducted by a large group of people working together to solve a problem is a form of peer-led approach [13]. Ideas, concepts, designs, and other elements collected from the group are synthesized into a final product that is presented to the community. It may present some advantages over traditional methods of public health intervention development. First, it is a bottom-up approach involving community members and peers instead of experts or academics that may lead to novel and relatable ideas regarding the topic at hand. Second, crowdsourcing increases community involvement and awareness of a specific issue or public health topic [14]. A systematic review found that crowdsourcing improved the quality, cost, and efficiency of research projects while engaging a large part of the public to create innovative materials [15]. Crowdsourcing is a popular approach in developing HIV interventions and has been shown to be feasible in engaging community members and in gathering culturally and locally relevant material [16].

While the intervention will be developed for young adults in general, we are interested in examining whether its effectiveness will differ between young adults enrolled in different types of tertiary educational institutions, which may vary in academic level and socioeconomic status. Open Online Courses were previously shown to be favorable towards higher educated (university educated) individuals [17, 18]. However, the study by Goldberg et al. [19] revealed that participants without a university degree were just as likely to complete the course and engage in online discussions. Significant improvements in outcomes were also found in lower educated participants as compared to higher educated participants in patient centered care or school-based interventions [20, 21]. A review showed that the effectiveness of health interventions varied between educational levels due to differences in the target focus: lower-educated individuals focused on knowledge acquisition while higher-educated individuals focused on behavioral change techniques. Hence, it is possible that our intervention is more effective and acceptable to those who are highly educated, but it is also possible that the material and teaching method has a higher impact for those who are less academic.

The main aim of this study is to promote the safe usage of dating applications among young adults in Hong Kong. We will conduct a cluster randomized controlled trial (RCT) to evaluate the effectiveness of a peer-led web-based intervention among young adults enrolled in three tertiary educational institutions. The specific objectives of the intervention are to: (1) provide appropriate and reliable information about dating applications; (2) increase the awareness of dating application associated risks and benefits; (3) improve the attitudes towards risk perception and the safe usage of dating applications; and (4) promote behavioral skills related to the safe usage of dating, specifically in preventing and handling negative experiences associated with dating applications. A post-hoc analysis will be conducted to identify factors that may affect the effectiveness of the intervention, which will then provide valuable insights to how interventions can be tailored for these young adults.

## Methods

### Study design

**Fig. 1:**
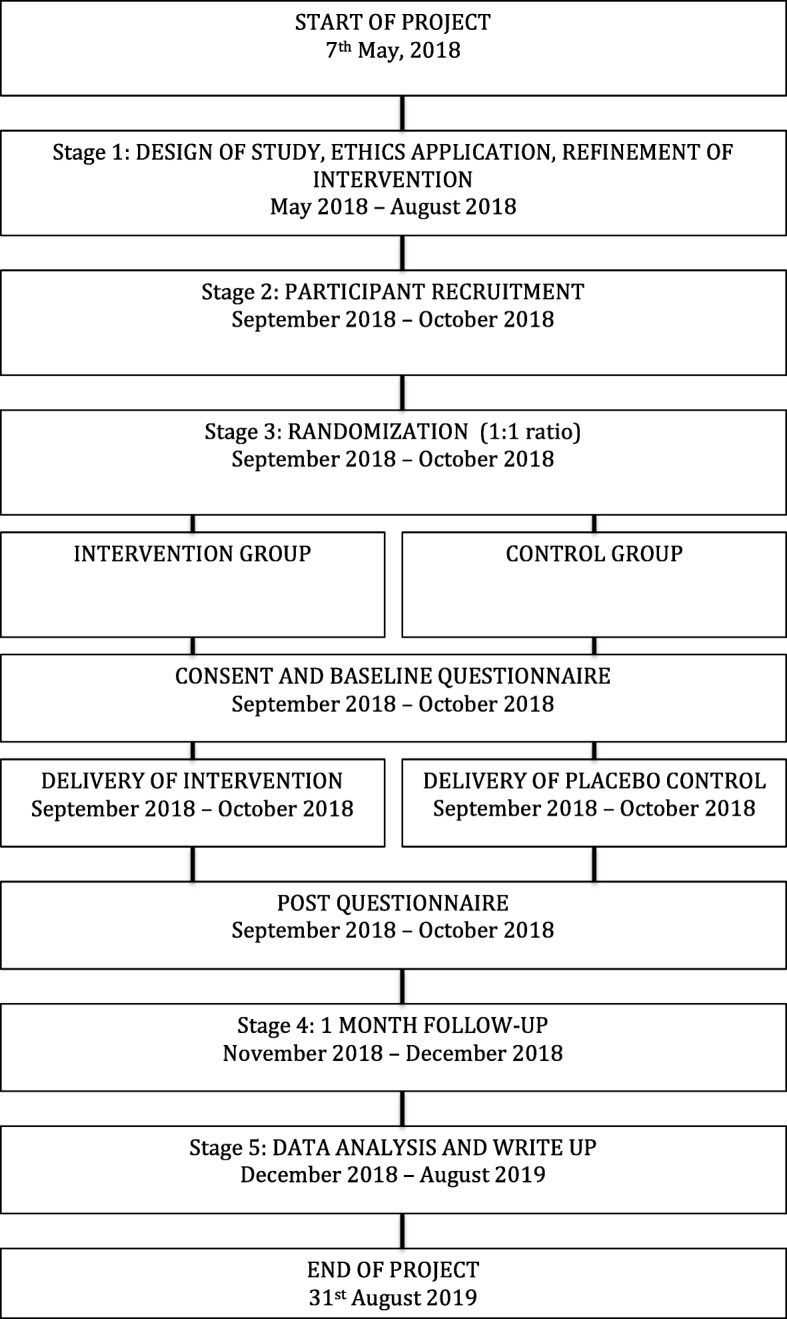
Study *flow chart*

**Fig. 2:**
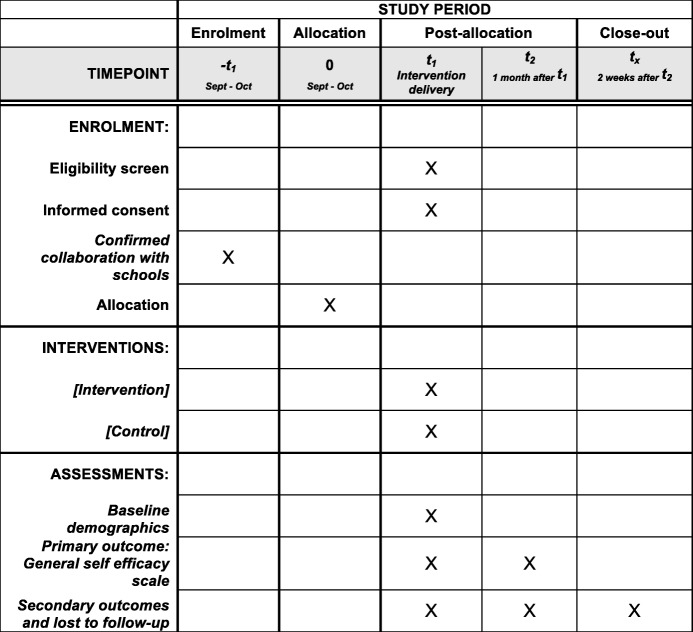
*Spirit figure* of study timeline

The study will use an open-labeled parallel cluster RCT design with one intervention arm and one placebo control arm with an allocation ratio of 1:1. The cluster unit will be by class. Individuals in both arms will complete the study instruments immediately before (pre-questionnaire) and after the intervention (post-questionnaire) and again one month after the intervention (follow-up questionnaire) (Figs. 1 and 2). There will not be any blinding as this is an open-labeled study, but class clusters will be randomly assigned to the intervention or control arm using randomization software and allocation concealment will be done utilizing the sealed envelope method [22]. An independent researcher will be in charge of generating the random allocation sequence and assigning the subject clusters to the intervention or control arms while the school will be responsible for enrolling the students into the study.

### Participants and eligibility criteria

Our target population is young adults aged 17–27 years recruited from three tertiary educational institutions in Hong Kong. This age range is selected as it is a critical period in the development and formatting of sexual attitudes and behaviors [23]. In order to be eligible to participate, the participants must also able to read and understand Chinese. Those with a physical impediment (e.g. blindness) preventing them from accessing the content will be excluded. Our research team will collaborate with school staff to ensure that these participants are excluded in an appropriate and sensitive manner.

### Study setting and recruitment

The three tertiary educational institutions are described below:

1. The Yijin Program is offered at seven educational institutions providing an alternative pathway for individuals who have completed high school or adult learners aged ≥ 21 years. In this one-year program, students would gain generic skills and knowledge including language, information technology, mathematical, interpersonal, and communication skills. The Yijin qualification is accepted by government agencies as meeting the academic entry requirement for civil service posts such as customs officer, fireman, tax inspector, or police officer [24]. However, the Yijin Diploma may not be used to apply directly to universities as it is not regarded as equivalent to the Hong Kong Examinations and Assessment Authority Examination.
2. Hang Seng Management College (HSMC) is a non-profit and self-financed private university institution offering both undergraduate and taught postgraduate degree programs. HSMC has five schools in the disciplines of Business, Communication, Decision Sciences, Humanities and Social Science, and Translation. HSMC utilizes a “liberal and professional” education model aimed to be a leading private liberal arts university in Asia. For 2017, its acceptance rate to Year 1 programs was 21% with the average Hong Kong Examinations and Assessment Authority score of 17 out of a total score of 49 [25].
3. The University of Hong Kong (HKU) is a public research university and is the oldest tertiary educational institution in Hong Kong. The university is organized into 10 academic faculties covering a diverse range of subjects and offers Bachelor degrees, Master degrees, and Doctorate degrees. HKU is regarded as one of the most prestigious universities in Asia, with five out of six applicants admitted being in the top 10% achievement ranking in the Hong Kong Examinations and Assessment Authority public examination [26].

The colleges and the teachers will recruit students to participate in the study and the intervention delivery will be carried out in one of the common core curriculum or soft skill classes.

### Sample size calculation

The sample size calculation is calculated for the primary outcome of self-efficacy. A cluster RCT regarding a school-delivered rights-based sexuality education for adolescents revealed a standard deviation of 0.56 and effect size of 0.20 for the outcome of protective self-efficacy [27]. With a minimal cluster size of 10 students, a 0.05 level of significance, power of 0.8, and intracluster correlation coefficient of 0.007, 260 participants and, with a 30% attribution rate, 338 students were estimated [27].

### Intervention development

The web-based intervention was developed with content and themes generated from focus group discussions (FGDs), followed by a crowdsourcing contest and further design workshop run by peer educators trained by StickyRiceLove, a local non-governmental organization (NGO) providing safe sex information to adolescents. Four FGDs of 25 young adults were first conducted to acquire issues related to dating applications. Results from FGDs were analyzed using the Grounded Theory with an open coding approach to generate themes and subthemes. Further intervention content materials were generated by a crowdsourcing contest entitled “Hi, Stranger! Dating Apps Education Design Contest” and elicited submissions of multimedia material such as videos, texts, images, and games to address the risks and benefits of dating application usage. The contest was promoted to a network of local NGOs focusing on sexual education and adolescent health and to students enrolled in a common core course on sexuality and culture at HKU. A total of 24 submissions were evaluated on the four elements of: content, interactivity, creativity, and credibility Four major domains were identified: sexual health; security of personal privacy; sexual harassment; and monetary scams. In the final development stage, a full-day workshop was held where StickyRiceLove peer educators synthesized the results from FGDs and crowdsourcing contest and used a Peer-Vetted Creative Production (PVCP) approach to develop the content and modes of delivery of the intervention [28].

### Intervention

The final intervention consists of four short videos with points of discussion, a scenario game, and a risk assessment tool. The short videos of 2–4 min in duration aimed to address the risks and benefits of using dating applications and encourage the viewers to reflect on their perception of dating application use in contrast with real-life scenarios. The videos cover the following in Additional files 1, 2, 3 and 4.


Additional file 1:**Video 1** is on the similarities between meeting people on dating applications and in real life. The video presents examples of being misled by dating profile characteristics and monetary scams.
Additional file 2:**Video 2** is on privacy concerns while using dating applications highlighting how one may be less alert when speaking to a stranger on applications because of the false sense of intimacy with high volumes of exchange of conversation.
Additional file 3:**Video 3** is on risky sexual behaviors while users scroll through multiple profiles and feel that they have found the “optimal” match which lead them to engage in risky sexual behavior.
Additional file 4:**Video 4** is on the legal issues and risk of sexual assault. This video provides information on the steps to take following a negative experience and the available resources.


#### Scenario game

The scenario game is a first-person simulation game where the players are presented with multiple choices when faced with different scenarios. Players first create a profile for themselves with characteristics such as sexual orientation, gender, and type of relationship one is looking for. The players’ choices will generate a dating application profile and a chat bubble will pop up with a message from a virtual matched partner. Players are then presented with 3–4 response choices below the chat box. The scenario game was designed with various algorithms dependent on the player’s choices to result in both positive and adverse outcomes. The final page will display a brief of why the cumulation of choices resulted in this outcome. The scenario game raises awareness on the potential consequences of dating application usage and promotes risk perception among players.

### Risk assessment tool

The risk assessment tool consists of 14 questions and will give the participant a score to infer on their risk level for adverse events (AEs) when using dating applications. The risk assessment tool addresses the five domains of sexual harassment, privacy concerns, monetary concerns, legal issues, and mental wellbeing.

### Control

The control will be an online activity that provides generic health advice using similar modalities (videos and interactive games). The control group will be asked to use the “Health exercise for all” campaign website by the Hong Kong Government [29]. It provides information for exercises suitable for all ages and at different settings in the form of informational pamphlets, instructional videos, and interactive games [29]. The campaign includes nine virtual classes with points of note and exercise guide and a set of videos of exercise demonstrations in the range of 20 s to 10 min. The website provides eight different interactive games such as “Calorie Restaurant,” “Do I have a big tummy,” and “I want to be more active.” The “Energy Expenditure Virtual Classroom” game will be utilized for the control group. The interactive game allows players to input personal statistics regarding their weight, gender, sports preference, and time allocated to each sport. The game will then produce a personalized training diary and offer exercise tips and other training recommendations.

### Delivery procedure

An investigative team member will attend a class to introduce the study and obtain written consent. Participants will be notified that participation is voluntary and that their refusal to participate will not affect their grades. Participants are allowed to withdraw from the study at any point in time without any reason. Participants are given a $50 HKD Coffee Coupon upon completion of the study.

Immediately before commencing the intervention, participants will complete an online pre-questionnaire and the risk assessment tool on their mobile device of choice such as smart phones, computers, or tablet. Four short videos will then be shown followed by a brief discussion on the issue presented in each video. There will be 15 min designated to play the scenario game or the control game before a post-questionnaire and the follow-up questionnaire at one month after the intervention. Students who do not complete the follow-up questionnaire after one week will be sent a reminder email. The follow-up questionnaire will be closed after two weeks of delivery and students who do not complete it will be taken into account at data analysis according to the intention-to-treat principle.

### Data management

The questionnaires will be constructed and administered using a password-secured survey tool called Survey Gizmo. The questionnaire responses will be automatically saved and exported to SPSS. All data from the study will be stored in a password-encrypted computer only accessible to members of the investigative team. The Patient Information and Consent Form (Additional file 5) will contain information for the participants regarding their rights to their private information and its collection, custody, retention, management, and use under Hong Kong Law. Participants are given contact information in the case of any AEs. All AEs will be recorded and the issues will be discussed with the investigative members on how it should be handled.

## Evaluation

### Theoretical framework

The Information, Motivation, and Behavioral Skills (IMB) model will be used to guide the evaluation of the intervention. This theoretical framework was used to guide the development of the pre-questionnaire and follow-up questionnaire. The IMB model states that behavioral change occurs as a result of information consisting of facts, heuristics, and implicit theories, motivations consisting of personal and social motivations, and behavioral skills consisting of self-efficacy and objective skills [30].

### Measurement variables

The pre-questionnaire contains baseline demographics and questions concerning dating application usage (Additional file 6). Demographic characteristics include participant’s age, gender, sexual orientation, relationship status, and housing situation. Dating application characteristics include purpose of dating application usage, types of applications used, and any AEs. Immediately after the intervention delivery, participants will fill out a post-questionnaire regarding the usability and appeal of the intervention (Additional file 7). Participants will be asked questions such as whether the intervention was interesting, if the content was appropriate, and if they would recommend the intervention to their peers. The follow-up questionnaire delivered one month after the intervention delivery will have matched questions with the pre-questionnaire and additional questions regarding dating application usage (Additional file 8).

The primary outcome will be the self-efficacy on the safe usage of dating applications among young adults in Hong Kong. This will be measured by the General Self Efficacy Scale [31]. Secondary outcomes include change of awareness in dating application associated risks and benefits, behavioral skills, attitudes towards risk perception, and outcomes from the validated scales of the Risk Propensity Scale [32] and the Patient Health Questionnaire-2 (PHQ-2) [33]. The Risk Propensity Scale is a nine-item scale measuring general risk-taking tendencies. Each item is rated on a 9-point Likert scale with a higher score indicating higher risk-taking tendencies [32]. The General Self Efficacy Scale is a 10-item scale measuring one’s self-belief in completing certain tasks or overcoming difficulties. Responses are marked on a 4-point Likert scale with a final score in the range of 10–40 points [31]. The PHQ-2 is a two-item scale used to evaluate depression symptoms [33].

### Statistical analysis

Data will be entered, cleaned, and analyzed using SPSS (Version 25). Target population demographics will be analyzed with descriptive statistics; comparison of baseline demographic characteristics between the intervention and control group will be conducted using *chi*-squared tests or t-tests. For outcome evaluation, paired t-tests will be used to examine within group outcome changes for continuous variables. McNemar’s test will be used to evaluate categorical outcomes. For the primary outcome, linear regression will be used to evaluate the factors between the intervention and control groups. Multilevel linear regression will be used to evaluate the differences in outcome between the intervention and control group and to account for interdependence of results due to the clustering effect. Non-conditional logistic regression will be used to identify factors that may affect the effectiveness of the intervention.

## Discussion

It is expected that this study will generate a breadth of information from both the development and evaluation of the intervention. The development process integrated methods that were shown to be effective in other health promotion initiatives [16, 34, 35] and this study will determine whether it is effective for the promotion of safe dating application usage. Successful aspects of the intervention can act as areas of further development for the investigative team and other NGOs. For example, favorable responses to the short videos can spur the development of content with similar style and length. Furthermore, the usage of the intervention can inform health researchers on the acceptability of online-based health interventions in young adults and can provide guidance on the development of similar peer-led interventions in the future.

There are several limitations that should be considered in this study. In the focus groups, promotion was done through the research team’s network of NGOs. While this allows the research team to target a group of people who may have an interest in this topic, it may overlook other individuals who belong to other NGOs or are not involved with NGOs at all. Promotions for the crowdsourcing contest were done to a common core course in The University of Hong Kong and the network of partner NGOs, once again resulting in a selective group of individuals. Since this cluster RCT is performed in a specific population, its results may not be generalizable to the public or in other countries.

In planning the evaluation of the intervention, we were concerned about several issues that could affect the validity of the study results. First, there is the possibility of contamination whereby individuals from the control and intervention groups could potentially interact with one another. The researchers will take great care to emphasize to participants during the introductory session that that participants should not discuss the content with other students until the end of the study. Participants will also be asked whether they have been exposed to other relevant content in the last month in the follow-up questionnaire. The cluster RCT design, with the class as clustering units, will also mitigate this potential problem by limiting the discussion of the intervention between study participants. Second, there is the potential of social desirability bias because all measures in the questionnaires are self-reported. We hope to minimize this bias by using an official secure survey tool and assuring participants that all their information is kept strictly confidential.

It is our hope that the intervention will reduce the risks associated with dating application usage and will be an interesting and well-rounded resource for young adults in Hong Kong. While this intervention is delivered in a classroom setting in this study, the entirety of the intervention content is web-based and can be accessed for free at the convenience of the public. This gives the intervention great potential to be widely disseminated and shared by the public. We hope that NGOs and other educational institutions will utilize this program as a resource for young adults seeking to learn more about safe and positive dating application usage. The usage of dating applications has been seen as a taboo in Hong Kong society and may have discouraged people from openly speaking about their experience or to share helpful tips in staying safe. However, the popularity of dating applications cannot be ignored; the creation and dissemination of this intervention could raise awareness of the risks and benefits of using dating applications and encourage discussion around this topic.

## Trial status

The study was approved by the Institutional Review Board of The University of Hong Kong/Hospital Authority Hong Kong West Cluster with IRB reference number UW 18–369. This protocol was registered in ClinicalTrials.gov with ID NCT03685643 (retrospectively registered) and also prospectively registered in the University of Hong Kong Clinical Trials Registry with ID HKUCTR-2512.

## Additional files

Additional file 5: Subject Information and Consent Form. (DOCX 70 kb)

Additional file 6: Pre-questionnaire. (DOCX 35 kb)

Additional file 7: Post-questionnaire. (DOCX 21 kb)

Additional file 8: Follow-up questionnaire. (DOCX 32 kb)

Additional file 9: SPIRIT Checklist. (DOC 122 kb)

## Abbreviations

AEs: Adverse Events
FGS: Focus Group Discussions
HKU: The University of Hong Kong
HSMC: Hang Seng Management College
IMB: Information Motivation Behavior model
IRB: Institutional Review Board
MSM: Men-who-have-sex-with-men
NGO: Non-governmental organization
PHQ-2: Patient Health Questionnaire-2
PVCP: Peer-Vetted Creative Production
