# Supplementary material for: Study protocol for a peer-led web-based intervention to promote safe usage of dating applications among young adults: a cluster randomized controlled trial
Source: Trials. 2019 Feb 6;20:102. doi: 10.1186/s13063-018-3167-5 (PMC6364485; doi:10.1186/s13063-018-3167-5)
Supplement: Supplementary file 5 — Subject Information and Consent Form. (DOCX 70 kb) [file 13063_2018_3167_MOESM5_ESM.docx]

**Appendix A**

**Subject Information and Consent Form**

**July, 2018 (Version 3. Dated 03.07.2018)**

The participants will be given a copy of the subject information form

**Evaluation of a peer-led web-based intervention for safe usage of dating applications in Hong Kong: a randomized controlled trial**

*You are being invited to take part in a research study. Before you decide it is important for you to understand why the research is being done and what it will involve. Please take time to read the following information carefully. Ask us if there is anything that is not clear or if you would like more information. Take time to decide whether or not you wish to take part.*

**Background and Aim**

With the improvement of mobile technology and reliance on smartphones, the use of dating applications has become very popular. Dating applications users are able to access a wide pool of potential partners that are geographically nearby. Many of the applications are free and lack strict joining criteria, resulting in a diverse range of users. At the same time, they could encourage the engagement in risky lifestyle behaviours, in addition to concerns regarding privacy and monetary scams. However, dating applications are also a promising way for one to meet new people and to find a romantic partner. Therefore, there is a need for appropriate resources regarding the safe usage of dating applications. An innovative and interactive online resource has been developed to educate young adults on this topic. The goal of this study to evaluate whether this resource is effective in promoting the safe usage of dating applications in Hong Kong.

**Participants**

Participants are young adults aged 17-27 years.

**Voluntary Participation**

It is up to you to decide whether or not to take part. If you do decide to take part you will be given this information sheet to keep and be asked to sign a consent form. If you decide to take part you are still free to withdraw at any time and without giving a reason. If you are in an educational institution, please be assured that your participation or withdrawal from the study will not have any effect on your study or grades at all.

**Procedures if you are to take part**

The study will last for a maximum of 3 months and will be a randomized controlled trial. The study design is done because sometimes because we do not know which way people learn best, we need to make comparisons. The groups are selected by a computer, which has no information about the individual. Participants will be divided into the program arm and control arm in a 1:1 ratio and then compared. One group will receive a website regarding dating applications and the other on active lifestyle. Both will be available to you afterwards.

You will first fill out a questionnaire that takes approximately 10 minutes. The program will consist of some text content, videos, and games. You will be given approximately 45 minutes to access the data. You will then fill out a post-intervention questionnaire, which will also last 2 minutes.

There are no lifestyle or dietary restrictions and there is a negligible chance of adverse effects resulting from the program. Please try to refrain from discussing the program with other people until the study is over.

**Potential side effects**

The program does not involve any medical treatment or drugs; hence the chance of any adverse health effects is negligible. You may experience some psychological distress because it brings up sensitive topics such as sexual behaviour and personal safety. However, all materials have been developed and validated by peers, sexual health experts, and NGO workers to ensure that it is appropriate. You can be assured that all information collected will be kept strictly confidential. A dedicated research staff member is available if you have any questions or concerns.

**Benefits of taking part of this study**

The program hopes to be a reliable and interesting resource on the topic of dating applications or lifestyle. Potential benefits include increasing one’s awareness of risks and benefits and improved behavioural skills. However, this cannot be guaranteed. This is the reason why your participation would be most helpful in allowing us to see whether our program actually works which may help us to develop better programs regarding this topic in the future.

**What happens when the study ends?**

The program will remain online and free for all to access after the end of the study. Please feel free to recommend this program to your friends.

**Confidentiality**

All information which is collected about you during the course of the research will be kept strictly confidential. Data will be securely stored in a password-encrypted computer.

You have the rights of access to personal data and publicly available study results, if and when needed.

Under the laws of Hong Kong (in particular the Personal Data (Privacy) Ordinance, Cap 486), you enjoy or may enjoy rights for the protection of the confidentiality of your personal data, such as those regarding the collection, custody, retention, management, control, use (including analysis or comparison), transfer in or out of Hong Kong, non-disclosure, erasure and/or in any way dealing with or disposing of any of your personal data in or for this study. For any query, you should consult the Privacy Commissioner for Privacy Data or his office (Tel No. 2827 2827) as to the proper monitoring or supervision of your personal data protection so that your full awareness and understanding of the significance of compliance with the law governing privacy data is assured.

By consenting to participate in this study, you expressly authorize:

- The principal investigator and his research team and the ethics committee (Institutional Review Board of the University of Hong Kong / Hospital Authority Hong Kong West Cluster) responsible for overseeing this study to get access to, to use, and to retain your personal data for the purposes and in the manner described in this informed consent process; and
- The relevant government agencies (e.g. the Hong Kong Department of Health) to get access to your personal data for the purposes of checking and verifying the integrity of study data and assessing compliance with the study protocol and relevant requirements.

**What will happen to the results of the research study?**

The results will be used for academic publications produced by the investigative team. All identifying data of the participants will be removed for publication. The results of the study may also be helpful in the future development of related resources.

**Funding and Review of the Study**

This study was funded by the Health Care and Health Promotion Fund (reference number: 8150355) and reviewed and approved by the Institutional Review Board of the University of Hong Kong /Hospital Authority Hong Kong West Cluster.

**Contact information**

If you have any concerns or questions regarding the study, you may contact Miss Stephanie Lau at [slau7@hku.hk](mailto:slau7@hku.hk) or 2518-5656.

On the following page, please read items 1, 2, and 3 and mark your initials in the box.

Please sign the following page, thank you so much for your participation!

**Subject Consent Form**

Centre Number:

Study Number:

Patient Identification Number for this trial:

**Title of Project: Evaluation of a peer-led web-based intervention for safe usage of dating applications in Hong Kong: a randomized controlled trial**

**Name of Researcher: Dr. William Wong**

|  | **Please initial box** |
| --- | --- |
| 1. I confirm that I have read and understood the information sheet dated ___/___/___ for the above study and have had the opportunity to ask questions. | ❒ |
| 2. I understand that my participation is voluntary and that I am free to withdraw at any time, without giving any reason, without my studies or legal rights being affected. | ❒ |
| 3. I agree to take part in the above study | ❒ |

_________________________ __________________ __________________

Name of subject Date Signature

_________________________ __________________ __________________

Name of witness (if applicable) Date Signature
