# Supplementary material for: Study protocol for a peer-led web-based intervention to promote safe usage of dating applications among young adults: a cluster randomized controlled trial
Source: Trials. 2019 Feb 6;20:102. doi: 10.1186/s13063-018-3167-5 (PMC6364485; doi:10.1186/s13063-018-3167-5)
Supplement: Supplementary file 6 — Pre-questionnaire. (DOCX 35 kb) [file 13063_2018_3167_MOESM6_ESM.docx]

**Appendix B: Pre-questionnaire**

Thank you for being a participant last month in this study for the University of Hong Kong aiming to promote the safe usage of dating applications. To further learn about your experience, we would be grateful if you could fill out this follow-up survey. Please rest assured that your response would be kept strictly confidential. Thank you for your time.

**Please enter your personal email: ____________________**

**A. Demographics**

A1. What is your current age? _______

A2. What is your gender?

1❒Male

2❒Female

A3. What is your sexual orientation?

1❒Heterosexual

2❒Homosexual

3❒Bisexual

A4. What is your relationship status?

1❒Single and not in a relationship

2❒In a relationship

3❒Married/co-inhabited

4❒Others (Please specify) ____________

A5. What type of housing do you currently live in?

1❒Public rental housing

2❒Housing Authority subsidized

3❒Private permanent housing

4❒Temporary housing

5❒Student dorm

6❒Village house

**B. Dating application usage**

B1. Have you ever used dating applications, instant message applications, or other social media platforms to meet new people?

1❒Yes

2❒No (please proceed to B6)

B2. Which applications have you used to meet new friends? (Tick all that apply)

1❒Heterosexual dating applications (Tinder, Coffee meets Bagel, Skout, Telegram)

2❒Homosexual dating applications (Grindr, Jack’d, Butterfly)

3❒Social media platforms (Instagram, Facebook)

4❒Instant messaging applications (Whatsapp, Wechat)

5❒Others (Please specify) _______

B3. What were your reasons for using these applications? (Tick all that apply)

1❒ Just out of boredom or curiosity

2❒ To meet new friends

3❒To meet other people with similar interests

4❒To find casual sex

5❒To find a romantic partner

B4. Please indicate your level of agreement on the follow statements in regards to dating application usage

|  | Strongly disagree | Disagree | Agree | Strongly agree |
| --- | --- | --- | --- | --- |
| 1. I spend more time using dating applications than I planned to | 1❒ | 2❒ | 3❒ | 4❒ |
| 2. Dating application usage has affected my school grades or work performance | 1❒ | 2❒ | 3❒ | 4❒ |
| 3. Dating application usage has affected my sleep | 1❒ | 2❒ | 3❒ | 4❒ |
| 4. Dating application usage as affected my relationships with my family members and/or friends | 1❒ | 2❒ | 3❒ | 4❒ |

B5. While using dating applications, have you encountered any of the situations below? (Tick all that apply)

1❒Scam or cheated

2❒Cyber-bullied or blackmailed

3❒Divulged personal information

4❒Pressured into sending intimate photos

5❒Stalking or secretly recorded by someone met online

6❒Was secretly recorded by someone met online

7❒Sexually harassed

8❒Sexually assaulted by someone met online

9❒None of the above

B6. Please indicate your level of agreement on the follow statements in regards to risk taking:

|  | 1 Strongly disagree | 2 | 3 | 4 | 5 | 6 | 7 Strongly agree |
| --- | --- | --- | --- | --- | --- | --- | --- |
| 1. Safety first. | 1❒ | 2❒ | 3❒ | 4❒ | 5❒ | 6❒ | 7❒ |
| 2. I do not take risks with my health. | 1❒ | 2❒ | 3❒ | 4❒ | 5❒ | 6❒ | 7❒ |
| 3. I prefer to avoid risks. | 1❒ | 2❒ | 3❒ | 4❒ | 5❒ | 6❒ | 7❒ |
| 4. I take risks regularly. | 1❒ | 2❒ | 3❒ | 4❒ | 5❒ | 6❒ | 7❒ |
| 5. I really dislike not knowing what is going to happen. | 1❒ | 2❒ | 3❒ | 4❒ | 5❒ | 6❒ | 7❒ |
| 6. I usually view risks as a challenge. | 1❒ | 2❒ | 3❒ | 4❒ | 5❒ | 6❒ | 7❒ |
|  | Risk avoider |  |  |  |  |  | Risk seeker |
| 7. I view myself as a…. | 1❒ | 2❒ | 3❒ | 4❒ | 5❒ | 6❒ | 7❒ |

**C. Information on dating application usage**

C1. Where do you get MOST of your information on dating applications? (Please tick one)

1❒Family/parents

2❒Friends

3❒Teachers/school staff

4❒Internet/online resource

5❒Magazines or other non-academic publications

6❒NGOs

C2. Please indicate how reliable you think this source of information is below:

1❒Unreliable

2❒Relatively reliable

3❒Reliable

**D. Behavioral skills in dating application usage**

D1. Please indicate your level of agreement on the follow statements in regards to dating application usage

|  | Not at all true | Hardly true | Moderately true | Exactly true |
| --- | --- | --- | --- | --- |
| 1. I can always manage to solve difficult problems if I try hard enough | 1❒ | 2❒ | 3❒ | 4❒ |
| 2. If someone opposes me, I can find the means and ways to get what I want | 1❒ | 2❒ | 3❒ | 4❒ |
| 3. It is easy for me to stick to my aims and accomplish my goals | 1❒ | 2❒ | 3❒ | 4❒ |
| 4. I am confident that I could deal efficiently with unexpected events | 1❒ | 2❒ | 3❒ | 4❒ |
| 5. Thanks to my resourcefulness, I know how to handle unforeseen situations | 1❒ | 2❒ | 3❒ | 4❒ |
| 6. I can solve most problems if I invest the necessary effort | 1❒ | 2❒ | 3❒ | 4❒ |
| 7. When I am confronted with a problem, I can usually find several solutions | 1❒ | 2❒ | 3❒ | 4❒ |
| 8. If I am in trouble, I can usually think of a solution | 1❒ | 2❒ | 3❒ | 4❒ |
| 9. I can usually handle whatever comes my way | 1❒ | 2❒ | 3❒ | 4❒ |
| 10. I can remain calm when facing difficulties because I can rely on my coping abilities | 1❒ | 2❒ | 3❒ | 4❒ |

D2. Over the past 2 weeks, how often have you been bothered by any of the following problems?

|  | Not at all | Several days | More than half the days | Nearly everyday |
| --- | --- | --- | --- | --- |
| 1. Little interest or pleasure in doing things | 1❒ | 2❒ | 3❒ | 4❒ |
| 2. Feeling down, depressed or hopeless | 1❒ | 2❒ | 3❒ | 4❒ |
