# Supplementary material for: Study protocol for a peer-led web-based intervention to promote safe usage of dating applications among young adults: a cluster randomized controlled trial
Source: Trials. 2019 Feb 6;20:102. doi: 10.1186/s13063-018-3167-5 (PMC6364485; doi:10.1186/s13063-018-3167-5)
Supplement: Supplementary file 7 — Post-questionnaire. (DOCX 21 kb) [file 13063_2018_3167_MOESM7_ESM.docx]

**Appendix C: Post-questionnaire**

Thank you for participating in our program a month ago. We hope that the content and delivery methods resulted in a useful and interesting product for you. Please take the time to fill out this form to help us get a better understanding of whether our program was beneficial and what could be improved. Thank you.

**Please enter your personal email: ____________________**

1. Please indicate your level of agreement with the following statements below:

|  | Strongly disagree | Disagree | Neutral | Agree | Strongly agree |
| --- | --- | --- | --- | --- | --- |
| 1. This program raised my interest in this topic | 1❒ | 2❒ | 3❒ | 4❒ | 5❒ |
| 2. The content covered is appropriate | 1❒ | 2❒ | 3❒ | 4❒ | 5❒ |
| 3. The program provided a sufficient mixture of explanation and practice | 1❒ | 2❒ | 3❒ | 4❒ | 5❒ |
| 4. The program developed my abilities and skills for the topic | 1❒ | 2❒ | 3❒ | 4❒ | 5❒ |
| 5. I will continue to use this program | 1❒ | 2❒ | 3❒ | 4❒ | 5❒ |
| 6. I would recommend this program to my friends | 1❒ | 2❒ | 3❒ | 4❒ | 5❒ |
|  |  |  |  |  |  |

2. Please identify what you consider to be ONE strength of the program.

________________________________________________________________

________________________________________________________________

3. Please identify ONE area where you think the program can be improved.

________________________________________________________________

________________________________________________________________

4. Please let us know if you have any other comments.

________________________________________________________________

________________________________________________________________
