# Supplementary material for: Study protocol for a peer-led web-based intervention to promote safe usage of dating applications among young adults: a cluster randomized controlled trial
Source: Trials. 2019 Feb 6;20:102. doi: 10.1186/s13063-018-3167-5 (PMC6364485; doi:10.1186/s13063-018-3167-5)
Supplement: Supplementary file 8 — Follow-up questionnaire. (DOCX 32 kb) [file 13063_2018_3167_MOESM8_ESM.docx]

**Appendix D: Follow-up Questionnaire**

Thank you for being a participant last month in this study for the University of Hong Kong aiming to promote the safe usage of dating applications. To further learn about your experience, we would be grateful if you could fill out this follow-up survey. Please rest assured that your response would be kept strictly confidential. Thank you for your time.

**Please enter your personal email: ____________________**

**A. Dating application usage**

A1. In the last month, have you ever used dating applications, instant message applications, or other social media platforms to meet new people?

1❒Yes

2❒No (please proceed to A6)

A2. In the last month, which applications have you used to meet new people? (Tick all that apply).

1❒Heterosexual dating applications (Tinder, Coffee meets Bagel, Skout, Telegram)

2❒Homosexual dating applications (Grindr, Jack’d, Butterfly)

3❒Social media platforms (Instagram, Facebook)

4❒Instant messaging applications (Whatsapp, Wechat)

5❒Others (Please specify) _______

A3. What were your current reasons for using these applications? (Tick all that apply)

1❒ Just out of boredom or curiosity

2❒ To meet new friends

3❒To meet other people with similar interests

4❒To find casual sex

5❒To find a romantic partner

A4. Please indicate your level of agreement on the follow statements in regards to dating application usage

|  | Strongly disagree | Disagree | Agree | Strongly agree |
| --- | --- | --- | --- | --- |
| 1. I spend more time using dating applications than I planned to | 1❒ | 2❒ | 3❒ | 4❒ |
| 2. Dating application usage has affected my school grades or work performance | 1❒ | 2❒ | 3❒ | 4❒ |
| 3. Dating application usage has affected my sleep | 1❒ | 2❒ | 3❒ | 4❒ |
| 4. Dating application usage as affected my relationships with my family members and/or friends | 1❒ | 2❒ | 3❒ | 4❒ |

A5. After our program, have you encountered any of the situations below while using dating applications? (Tick all that apply)

1❒Scam or cheated

2❒Cyber-bullied or blackmailed

3❒Divulged personal information

4❒Pressured into sending intimate photos

5❒Stalking or secretly recorded by someone met online

6❒Was secretly recorded by someone met online

7❒Sexually harassed

8❒Sexually assaulted by someone met online

9❒None of the above

A6. Please indicate your level of agreement on the follow statements in regards to risk taking:

|  | 1 Strongly disagree | 2 | 3 | 4 | 5 | 6 | 7 Strongly agree |
| --- | --- | --- | --- | --- | --- | --- | --- |
| 1. Safety first. | 1❒ | 2❒ | 3❒ | 4❒ | 5❒ | 6❒ | 7❒ |
| 2. I do not take risks with my health. | 1❒ | 2❒ | 3❒ | 4❒ | 5❒ | 6❒ | 7❒ |
| 3. I prefer to avoid risks. | 1❒ | 2❒ | 3❒ | 4❒ | 5❒ | 6❒ | 7❒ |
| 4. I take risks regularly. | 1❒ | 2❒ | 3❒ | 4❒ | 5❒ | 6❒ | 7❒ |
| 5. I really dislike not knowing what is going to happen. | 1❒ | 2❒ | 3❒ | 4❒ | 5❒ | 6❒ | 7❒ |
| 6. I usually view risks as a challenge. | 1❒ | 2❒ | 3❒ | 4❒ | 5❒ | 6❒ | 7❒ |
|  | Risk avoider |  |  |  |  |  | Risk seeker |
| 7. I view myself as a…. | 1❒ | 2❒ | 3❒ | 4❒ | 5❒ | 6❒ | 7❒ |

A7. In the past month, have you seen any other material related to dating applications?

1❒Yes

2❒No (please proceed to A9)

A8. Where did you see or access this material?

1❒The University of Hong Kong’s Dating Application Intervention

2❒Internet

4❒Magazines

5❒TV

6❒Social media

A9. After using the program, do you feel you are:

|  | Not at all true | Hardly true | Moderately true | Exactly true |
| --- | --- | --- | --- | --- |
| 1. Able to cope with using dating applications | 1❒ | 2❒ | 3❒ | 4❒ |
| 2. Able to understand your risks associated with dating application usage | 1❒ | 2❒ | 3❒ | 4❒ |
| 3. Confident about your abilities to use dating applications safely | 1❒ | 2❒ | 3❒ | 4❒ |
| 4. Able to protect yourself while using dating applications | 1❒ | 2❒ | 3❒ | 4❒ |

A10. Apart from the initial workshop in class, how many times have you visited the program in the past month?

1❒None 4❒4-5 times

2❒1-2 times 5❒Over 5 times

3❒3-4 times

A11. How many of your friends have your recommended the program to?

1❒None 4❒4-5 friends

2❒1-2 friends 5❒Over 5 friends

3❒3-4 friends

**B. Behavioral skills in dating application usage**

B1. Please indicate your level of agreement on the follow statements in regards to dating application usage

|  | Not at all true | Hardly true | Moderately true | Exactly true |
| --- | --- | --- | --- | --- |
| 1. I can always manage to solve difficult problems if I try hard enough | 1❒ | 2❒ | 3❒ | 4❒ |
| 2. If someone opposes me, I can find the means and ways to get what I want | 1❒ | 2❒ | 3❒ | 4❒ |
| 3. It is easy for me to stick to my aims and accomplish my goals | 1❒ | 2❒ | 3❒ | 4❒ |
| 4. I am confident that I could deal efficiently with unexpected events | 1❒ | 2❒ | 3❒ | 4❒ |
| 5. Thanks to my resourcefulness, I know how to handle unforeseen situations | 1❒ | 2❒ | 3❒ | 4❒ |
| 6. I can solve most problems if I invest the necessary effort | 1❒ | 2❒ | 3❒ | 4❒ |
| 7. When I am confronted with a problem, I can usually find several solutions | 1❒ | 2❒ | 3❒ | 4❒ |
| 8. If I am in trouble, I can usually think of a solution | 1❒ | 2❒ | 3❒ | 4❒ |
| 9. I can usually handle whatever comes my way | 1❒ | 2❒ | 3❒ | 4❒ |
| 10. I can remain calm when facing difficulties because I can rely on my coping abilities | 1❒ | 2❒ | 3❒ | 4❒ |

B2. Over the past 2 weeks, how often have you been bothered by any of the following problems?

|  | Not at all | Several days | More than half the days | Nearly everyday |
| --- | --- | --- | --- | --- |
| 1. Little interest or pleasure in doing things | 1❒ | 2❒ | 3❒ | 4❒ |
| 2. Feeling down, depressed or hopeless | 1❒ | 2❒ | 3❒ | 4❒ |
